# Functional Role of RBP in Osteosarcoma: Regulatory Mechanism and Clinical Therapy

**DOI:** 10.1155/2023/9849719

**Published:** 2023-06-30

**Authors:** Ziyuan Que, Kang Yang, Nan Wang, Shuying Li, Tao Li

**Affiliations:** ^1^Yangzhou University Medical College, Yangzhou University, Yangzhou 225009, Jiangsu Province, China; ^2^Department of Zhejiang Cancer Hospital, Hangzhou Institute of Medicine (HIM), Chinese Academy of Sciences, Hangzhou 310022, Zhejiang, China

## Abstract

Malignant bone neoplasms can be represented by osteosarcoma (OS), which accounts for 36% of all sarcomas. To reduce tumor malignancy, extensive efforts have been devoted to find an ideal target from numerous candidates, among which RNA-binding proteins (RBPs) have shown their unparalleled competitiveness. With the special structure of RNA-binding domains, RBPs have the potential to establish relationships with RNAs or small molecules and are considered regulators of different sections of RNA processes, including splicing, transport, translation, and degradation of RNAs. RBPs have considerable significant roles in various cancers, and experiments revealed that there was a strong association of RBPs with tumorigenesis and tumor cell progression. Regarding OS, RBPs are a new orientation, but achievements in hand are noteworthy. Higher or lower expression of RBPs was first found in tumor cells compared to normal tissue. By binding to different molecules, RBPs are capable of influencing tumor cell phenotypes through different signaling pathways or other axes, and researches on medical treatment have been largely inspired. Exploring the prognostic and therapeutic values of RBPs in OS is a hotspot where diverse avenues on regulating RBPs have achieved dramatical effects. In this review, we briefly summarize the contribution of RBPs and their binding molecules to OS oncogenicity and generally introduce distinctive RBPs as samples. Moreover, we focus on the attempts to differentiate RBP's opposite functions in predicting prognosis and collect possible strategies for treatment. Our review provides forwards insight into improving the understanding of OS and suggests RBPs as potential biomarkers for therapies.

## 1. Introduction

Osteosarcoma (OS), the most frequent occurrence in bone sarcomas, is the primary malignant neoplasm of mesenchymal tissue that occurs in the long bone metaphysis, especially in the distal femur and proximal tibia [1]. Stimulating data have concluded the significant impact of age on tumorigenesis and gender preference, in which boys above 13 years old have more preference to attack [2]. People in the sixth decade have a similar risk of affecting OS as those in the 20th, but surprisingly, elderly people have the same survival rates as the young [2]. Similar to other cancer cells, OS cells have a strong tendency in hematogenous metastasis, and the lung is the optimal organ for cells to regrow, which brings a sharp drop to a 5-year event-free survival (EFS). Ground-breaking achievements have been made by the combination of surgical operation, neoadjuvant and adjuvant chemotherapy, and radiotherapy in maintaining 5-year EFS to more than 70% with OS cells localized [3, 4]. Nevertheless, the growth of EFS reached a plateau in the last 30 years [5], and there are abundant challenges in improvement; in particular, less than half of patients who have metastatic tumors will not be disturbed by tumor recurrence in 5 years [2]. Under this circumstance, new strategies to improve the prognosis of OS are urgently needed.

Current studies focusing on the basic mechanism of cancer cell phenotypes, including proliferation, invasion, apoptosis, and epithelial-mesenchymal transcription (EMT), have gradually helped us uncover the mystery of cancer, and during the course, a vast number of proteins and molecules have been reported to have potential functions in influencing cancer cells. Among these proteins and molecules, RNA-binding proteins (RBPs) with multiple regulatory RNAs are considered participants impacting the cancer cell phenotypes and have become a new hotspot in this research area [6, 7]. Stimulating evidence has proven that aberrant expression of RBPs is linked to tumorigenesis [8, 9], and regulating RBP expression are possible approaches to cancer therapy. Herein, we conclude abnormal expression of RBPs, emphasize the combination of RBPs and multiple molecules in regulating cell phenotypes, and present prognostic and therapeutic values, suggesting that RBPs may be possible avenues in OS treatment.

## 2. Interactions of RBPs and RNAs

### 2.1. RBPs Bind to RNAs through RNA-Binding Domains

Identified mostly in plants, animals, and microbial species [10], RBPs were originally thought of as proteins binding to coding and noncoding RNAs through RNA-binding domains (RBDs) and formed a stable secondary structure. The classical viewpoint stated that RBDs taking part in ribonucleoprotein complexes were all well-defined, such as the RNA recognition motif (RRM), K homology (KH), DEAD box helicase domains, and others [11–15]. The consensus had been reached for a long time, but new findings that searched out the structure of large ribonucleoproteins consisting of RBPs with unknown RBDs forcefully challenged this point [16]. Advanced studies revealed that previous well-defined RBDs are classified as canonical RBDs and account for just a quarter of total RBDs, whereas other noncanonical RBDs are in the process of searching and concluding their structures and functions. For example, prion-like domain (PrLD) leads to liquid–liquid phase transitions but has a preference for RBP misfolding, which causes neurodegenerative disease [15]. Both canonical and noncanonical RBDs are reliable tools to connect with RNAs and naturally affect RNA metabolism.

### 2.2. RBPs Regulate RNAs at the Posttranscriptional Level

Apart from its incredible function in forming ribonucleic protein complexes, RBPs act as regulators in posttranscriptional processes, including alternative splicing, alternative polyadenylation, capping, modification, mRNA stabilization, and mRNA localization [17]. Alternative splicing is the major regulation that promotes protein diversity and mRNA stability, and RBPs are determinants for alternative splicing. Abnormal RBPs are partly due to the disturbed splicing process and induce tumor growth [18]. Alternative polyadenylation is utilized to explain the work of generating transcripts and modifying the coding sequence to impact the protein [19]. RNA stability is closely related to its poly(A) tail at the 3′ end, and some RBPs can stabilize the RNA structure by capping the RNA poly(A) tail with an N7 methylated guanine (m7GpppN) [20]. RBPs can also recognize cis motifs or zip codes in the 3′-untranslated region (3′-UTR) of RNA to regulate the localization of RNA in cells. RBPs are necessary to activate RNA translation in all stages of the progress [21]. More collective and sufficient evidence can be found in the previous review [6].

## 3. RBPs as Hub Regulators in Diverse Cancers

Aberrant expression or disorders of RBPs affect RNA metabolic processing and alter gene expression patterns, driving serious diseases or even cancers. To obtain more information on the fundamental mechanism, much attention has been given to this field. Ectopic expression of RBPs was first noticed in various tumor tissues, and among these tissues, elevated expression was more easily observed. Human RBP La has been demonstrated to be overexpressed in lung, cervical, head and neck, and chronic myelogenous leukemia, leading to various cancers by supporting proliferation, mobility, invasion, and maintaining the survival of cancer cells that may cause chemotherapeutic resistance [22]. Musashi (MSI) proteins were highly expressed in colorectal, lung, and pancreatic cancers, glioblastoma, and several leukemias by operating crucial oncogenic signaling pathways [23]. In addition to those highly expressed RBPs, low expression may also indicate high malignancy. One of the examples is polyC-RNA-binding protein 1 (PCBP1). As a member of the PCBP family proteins, PCBP1 is reported to be reduced in lung cancer, cervical cancer, breast cancer, colon cancer, and liver cancer [24–27], suggesting that altering PCBP1 expression may become a possible therapeutic strategy. Moreover, previous studies have elucidated the identification of RBPs as prognostic factors and treatment targets in cancers. For example, high expression of RNA-binding motif protein 38 (RBM38) represents longer overall survival in ovarian cancer, breast cancer, and glioma [28] but implies poor prognosis in breast cancer [29]. The opposite meanings indicate the dual role of RBM38 in predicting prognosis, and more roles of RBPs in the prognosis and potential therapy of cancers deserve to be explored in future studies.

## 4. Dysregulation of RBPs in OS

To date, RBPs and cancers are hotspots in investigating the primary mechanism of cancer progression, and OS is one of those investigated cancers that shows a considerable relationship with RBPs. Summarized from several studies, we identified several dysregulated RBPs in OS, and the correlated molecules were also collected (Table 1). From the expression level, four RBPs (RBM10, PUM2, QKI2, and TARBP2) were downregulated in tumor tissues compared with normal tissues, while other identified RBPs were consistently upregulated in OS cells.

## 5. RBPs Act as Oncogenic Proteins or Anticancer Proteins to Regulate OS Cells

**Figure 1:**
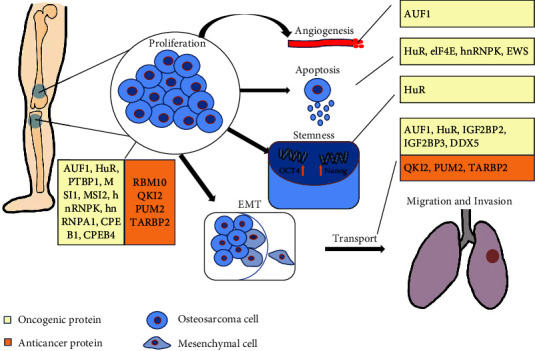
Diverse function of RBPs in OS progression.

Dysregulation of RBPs in OS is the most typical and exterior characteristic of all considerable functions in promoting or suppressing tumor cells, and the mechanism of RBP expression need further explorations. As shown in the stimulating data, RBPs have a significant impact on the junction with OS cell phenotypes, such as proliferation, migration and invasion, EMT, and apoptosis. We cartoon different roles of RBPs in OS progression (Figure 1). Of note, IGF2 mRNA binding protein 3 (IGF2BP3) is consistently regarded as an oncofoetal protein [60], and this assertion was strongly proven in murine OS cells [48]. Abundance analyses revealed that the expression of IGF2BP3 was higher in AXT cells than in AX cells, which show less malignancy than the former, and contributed to activating tumors and promoting tumor cell growth in vivo and in vitro. Human antigen R (HuR) was elucidated to be tightly correlated with OS cell migration and invasion, lying in supporting cells to abandon epithelial cell-like characteristics. However, when HuR was knocked down, this effect was attenuated [32]. In addition, DDX3 interacting with heterogeneous nuclear ribonucleoprotein K (hnRNPK) via its C-terminal region contributed to cell apoptosis, and further studies demonstrated that modified DDX3 lost the ability to initiate DNA damage progression due to unfavorable contact with hnRNPK, which emphasizes the credible efficiency of DDX3-hnRNPK to induce apoptosis [42].

RBPs and their binding molecules are regulators of each other, and the combinations act directly on tumor cell progression. Evidence below have shown that regulations on RBPs or their binding molecules can bring out wonderful effects to reduce tumor malignancy. To more specifically amplify the roles of RBPs in suppressing or supporting OS cell progression, conclusions on the interaction between RBPs and molecules will be shown via the classification of molecule types.

### 5.1. Interaction with Noncoding RNAs

#### 5.1.1. RBPs and MicroRNA (miRNAs)

miRNAs are highly conserved small noncoding RNAs that have been identified as suppressive effectors in regulating mRNAs and are critical for cellular progress and developmental pathways [61]. Collective analyses indicated that RBPs play irreplaceable roles in the canonical and noncanonical pathways to mature miRNAs. The double-stranded RBP DGCR8 and its cooperator RNase III-type enzyme DROSHA were observed to guide primary miRNA (pri-miRNA) into precursors miRNA (pre-miRNA) in the nucleus, which eventually turned into mature miRNA with the help of DICER in the cytoplasm. In the other pathways, miRNA expression was greatly influenced by RBPs in positive or negative effects through binding to the terminal loop of pri-miRNAs and pre-miRNAs [62]. Cooperation of miRNAs and RBPs has a considerable impact on regulating diseases and malignant tumors. For example, upregulated fused in sarcoma (FUS) and downregulated miR-138-5p were correlated with the regulation of angiogenesis with the mediator circ_002136 in glioma cells [63]. In this part, we gather the studies involving the interplay of miRNAs and RBPs in OS to emphasize the favorable effect on cancer regulation.

**Figure 2:**
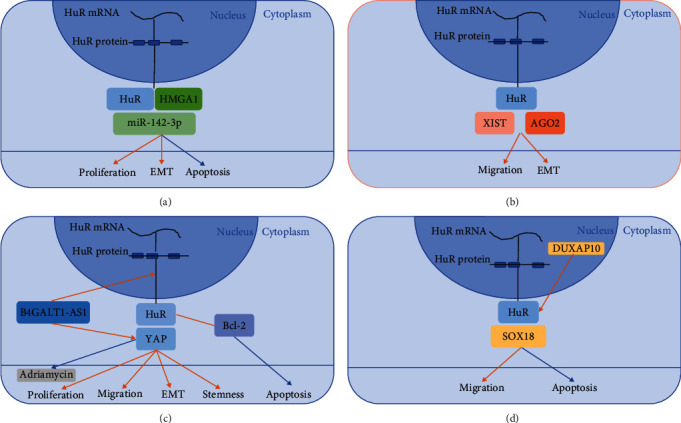
Mechanisms of HuR in regulating OS progression. Red arrows represent positive regulation, while blue ones are negative. (a) HuR binds to HMGA1 and miR-142-3p to promote OS cell proliferation and EMT while reduce cell apoptosis at the same time. (b) LncRNA XIST combined AGO2 and HuR to promote EMT and migration of OS. (c) HuR was positively related to Bcl-2 overexpression and thus reduce apoptosis. B4GALT1-AS1 was contributed to the delivery of HuR from nucleus to cytoplasm and upregulated YAP expression that promotes OS cell proliferation, migration and invasion, EMT, and stemness. The effect of Adriamycin was observed attenuated when YAP overexpressed. (d) DUXAP10 was largely expressed in nucleus and was demonstrated to bind HuR and SOX18 to promote migration and invasion and reduce apoptosis of OS cells.

HuR, also termed ELAVL1, is the only antigen to be expressed in all human tissues among all ELAVL family members [64] containing two tandem RRMs, a hinge region, and a third RRM [65], and the abundant proof was collected to completely explain the regulatory mechanism. Pan et al. [35] found that HuR could harbor high-mobility group AT-hook 1 (HMGA1) and that miR-142-3p could directly bind to the HMGA1 3′ untranslated region. With the characteristics of reducing miR-142-3p expression and increasing HMGA1 expression, HuR notably promotes OS cell progression by suppressing the miR-142-3p/HMGA1 axis (Figure 2(a)).

With two RRMs, AU-binding factor 1 (AUF1) is an RBP rooted in alternative premessenger RNA (pre-mRNA) splicing [66] and is involved in mRNA stability and mRNA translation [64]. In particular, AUF1 is mostly upregulated in cancers, including breast, skin, thyroid, and liver cancers [67, 68], as well as in OS. AUF1 was found to be strongly modulated by miR-141 and miR-146b-5p. The relationship between regulators and the effector was sensitive. AUF1 was shown to be correlated with the proliferation, migration, invasion, and mesenchymal features of OS cells but was reduced by miR-141 and miR-146b-5p [38].

PUM2 is a Pumilio (PUM) protein encoded by *PUM* genes on chromosome 2 containing a C-terminal highly conserved PUF domain that binds to several protein cofactors to repress direct mRNA and lead to degradation [69]. However, in another aspect, PUM2 has an effort to develop mammalian neural stem cells, epilepsy, and human germ cell progression [70–72], and accumulating evidence proving that PUM2 is correlated with cancer progression [73, 74]. As a tumor suppressor, PUM2 is typically attenuated in OS tissue. Hu et al. [41] designed a series of experiments to determine the significant repression of overexpressed PUM2 on OS cell proliferation, migration, and progression in vivo. Subsequent studies investigated whether PUM2 could regulate and interact with its potential target STARD13 by binding to the STARD13 3′-UTR with miR-590-3p and miR-9. With this binding, PUM2 exerts an inhibitory effect on OS cells.

Quaking (QKI), one of the signal transduction and activation of RNA (STAR) protein family and hnRNPK homology type family [75], is involved in posttranscriptional mRNA processing, including facilitating spliceosomal complex formation, mRNA stability, and localization [76–78]. There are backlogging certifications disclosing that QKI serves as a tumor suppressor gene in different types of cancers [79–81]. QKI2 is one of the isoforms of QKI, showing its surprising role in restraining OS cell progression. Yang et al. [43] demonstrated that the decrease in QKI2 protein expression was regulated by the miR-17-92 cluster in OS and led to a decrease in *β*-catenin protein levels soon afterward. Based on the discovery, they concluded that the miR-17-92 cluster/QKI2/*β*-catenin axis promotes OS progression. A few years later, they noticed positive characteristics of miR-20a in OS cell proliferation, migration, and invasion and found that QKI2 is a direct target of miR-20a [44]. QKI2 mRNA can be inhibited by aberrantly increased miR-20a and thus promote OS cell progression, implying that QKI2 has a potential suppressive influence in OS cells.

As a critical regulator of tumor and stem cells, the IGF2 mRNA-binding protein family (IGF2BPs) are oncogenic proteins in cancer [82] containing RRM and KH domains. In the consistent research, a consensus has been reached that IGF2BP1 has a strong conversed potential in cancer-derived cell lines [83, 84]. Mostly expressed in fetal tissues, while in a few adult tissue cases, IGF2BP1 is important in embryogenesis, carcinogenesis, and chemoresistance by influencing the stability, translatability, or localization of direct mRNAs [85, 86]. Consistently, IGF2BP1 is a prognostic factor in abundant human cancers with high expression in tumor tissue. Wang et al. [87] detected the expression levels of miR-150 and IGF2BP1 at the mRNA and protein levels and evaluated the associations of miR-150 and/or IGF2BP1 protein expression. With data analysis, they elucidated the imbalance of the miR-150- IGF2BP1 axis, which contributed to the development, progression, and prognosis of OS, leaving the mechanism problem of the miR-150- IGF2BP1 axis unsolved. Later, miR150 modulated with mesenchymal stem cell-derived exosomes was also verified to downregulate IGF2BP1 and suppress OS development [45].

Similar to the prion domain in yeast, PrLD is characterized by low complexity and exists in nearly 70 RBPs in humans [15]. FUS is an RNA- or DNA-binding protein that was originally detected in human liposarcoma [88] characterized with PrLD. Coupled with splicing regulators or precursor mRNAs, FUS is capable of regulating RNA splicing [89]. Aberrant mutation of FUS can frequently cause amyotrophic lateral sclerosis, a well-known neurodegenerative disease, and FUS aggression has been identified ubiquitously in other neurodegenerative diseases, such as frontotemporal lobar degeneration, polyglutamine diseases, essential tremor and Parkinson's disease [90–93]. Currently, the property of FUS in stabilizing the high expression of messenger RNA (mRNA) lactate dehydrogenase B (LDHB) was illustrated by Wang [50]. LDHB is unfavorably overexpressed in OS cells and can promote tumor malignancy. In a later study, upstream of FUS, miR-141-3p was identified to be downregulated in OS cells, and its upregulation was correlated with the abatement of FUS and LDHB at both the mRNA and protein levels.

The Ewing sarcoma breakpoint region one gene, also known as the Ewing sarcoma breakpoint region 1 (*EWSR1*) gene, is a member of the *FET* gene family (together with *FUS/TLS* and *TAF15*) and consists of a low-complexity PrLD and an SYGQ-rich N-terminal transactivation domain [94]. EWS was identified as having a critical impact on the pathogenesis of Ewing's sarcoma, and its regulation in OS was uncovered in later research. He and Ding [51] discovered that upregulated EWS was typically characterized in OS and was related to tumor size, advanced stage, and metastasis. The corresponding factor Sox2 was adjusted by EWS to induce OS cell proliferation, colony formation, and apoptosis. Subsequent analysis proved that miR-199a-5p could directly bind to the Sox2 3′-UTR and negatively affect Sox2 biological function in MG63 cells, indicating that EWS regulates OS cells through the miR-199a-5p/Sox2 axis.

DDX5 is typically recognized with an Asp-Glu-Ala-Asp (DEAD) motif [64] and belongs to the largest helicase family. Previous studies elucidated that DDX5 was highly expressed in OS and that multiple factors were involved in the modulation. Chen et al. [53] found that miR-671-5p was able to attenuate DDX5 expression and downregulate long noncoding RNA (lncRNAs) DLEU1 expression to deplete OS cell proliferation and migration. Subsequently, Mao et al. [54], inspired by previous research, identified miR-214-5p as having similar effects on DDX5, which was sponged by circ-XPR1 to induce OS cell progression.

RBPs of eukaryotic translation initiation factor 4E (eIF4E) are characterized by cap-binding domains and are well known to induce tumorigenesis and cancer progression. Qi et al. [55] inferred that eIF4E was negatively influenced by overexpressed miR-496, which was pervasively expressed at low levels in OS cells. Evidence has shown that miR-496 acts as an inhibitor of OS cell proliferation, migration and invasion, and suppresses tumor growth in vivo by depressing the expression of eIF4E, suggesting a potential therapeutic target for OS treatment.

Cytoplasmic polyadenylation element-binding protein (CPEB) is a series of binding proteins that regulate mRNA translation via the 3′-UTR of mRNAs [95], and CPEB1 has been considered a positive factor in tumorigenesis. Wang et al. [57] showed that CPEB1 was upregulated in OS and had a close relationship with OS cell proliferation and metastasis. In structure, the target gene *miR-320a* binds directly to the 3′-UTR of CPEB1 to indirectly inhibit the biological function of CPEB1 by downregulating its expression. In addition, Zhou et al. [58] enumerated another target gene, *miR-377-3p*, that binds to both CPEB1 and circ_0003732 and has meaningful effects on suppressing CPEB1 effectiveness.

#### 5.1.2. RBPs and lncRNAs

lncRNAs are a large group of RNAs that are candidates for multiple modifications of cancers through binding molecules. lncRNAs were demonstrated to sponge miRNAs and combine with RBPs to induce cancer progression [96] and control stem cell self-renewal and differentiation [97]. However, well-defined lncRNAs represent only a minor part of all lncRNAs, and more explorations of the foundations of lncRNA regulation remain to be carried out. In this section, we summarize the interplay of lncRNAs and diverse RBPs and/or miRNAs in modulating OS cell phenotypes and reveal the mechanism of RBPs and lncRNAs as much as possible.

The oncogenic role of the lncRNA XIST in OS was previously uncovered [98], but the exact mechanism remains unexplored. Liu et al. [31] verified the linkage of lncRNA XIST and RBP HuR as possible regulators of OS cell progression. Then, they discovered that AGO2 was positively related to mRNA to facilitate the activation of XIST-HuR on EMT and migration in OS cells, indicating that silencing AGO2 may become a possible way to reduce HuR [31] (Figure 2(b)). lncRNA B4GALT1-AS1 has a function inverse to B4GALT1 and was verified as a suppression factor for hepatic gluconeogenesis and lipogenesis [99]. Li et al. [33] discovered the process of HuR translocation from the nucleus to the cytoplasm by virtue of B4GALT1-AS1 and thus upregulated YAP expression, explaining the regulatory effect of B4GALT1-AS1 in promoting OS proliferation, migration, and cell stemness (Figure 2(c)). lncRNA double homeobox A pseudogene 10 (DUXAP10) is especially overexpressed in OS tissues and acts as a promotor of OS cells in vivo and in vitro. Wang et al. [34] demonstrated the correlation between DUXAP10 and HuR, and SOX18 acted as a downstream factor of DUXAP10 to affect OS cell progression (Figure 2(d)).

MSI2 is a translational repressor participating in regulating asymmetric division, hematopoietic, and intestinal systems, self-renewal, etc. [22, 100–103]. MSI2 is generally considered an oncogenic factor in promoting cancer cell proliferation, invasion, migration, and metastasis and predicting prognosis [104–106]. In addition, the function of MSI2 in regulating OS cells has been uncovered based on a deep comprehension of lncRNAs. Zhang et al. [37] proved the tumorigenesis influence of lncRNA anti-differentiating noncoding RNA (DANCR) in OS. Inspired by the correlation of DANCR and the miR-149/MSI2 axis in bladder cancer, they verified the combination of DANCR and miR-149 and the inverse correlation between MSI2 and miR-149. Thus, they concluded that DANCR can target OS cells by regulating the miR-149/MSI2 axis.

Polypyrimidine tract-binding protein 1 (PTBP1) participates in all aspects of the mRNA cascade, and its combination with lncRNAs was verified. Yao et al. [40] demonstrated that lncRNA HOPPIT had a positive correlation in booming PTBP1 expression and regulated KH-type splicing regulatory protein via PTBP1 to activate OS cell proliferation, migration, and invasion but left the limitation on therapy value for further research.

IGF2BP2 is generally considered a participant in the localization, stability, and translation of RNAs and is also capable of abating RNA endonucleases or miRNA-mediated degradation [107, 108]. Recent studies revealed that IGF2P2 was beneficial for inducing OS, suggesting an association between the dysregulation of IGF2BP2 and cancer progression. Gu et al. [46] verified the hypothesis that IGF2BP2 was recruited by lncRNA HCG11 to stabilize p27 Kip1 mRNA and helped HCG11 suppress human OS growth.

CPEB4 belongs to the CPEBs and serves as a promoter on OS because of its high expression in all types of OS cell lines [59]. Yang et al. [59] provided an assumption that the role of lncRNA RP11-361F15.2 was important in regulating OS cell progression by connecting and interacting with its target genes *miR-30c-5p* and CPEB4. The later experiments proved the inverse correlation of antitumour gene *miR-30c-5p* and oncogenes CPEB4 or lncRNA RP11-361F15.2, which exploited updated approaches to repress OS cell growth and invasion.

La-related protein 1 (LARP1) is a member of the LARP family with a highly conserved La module. It is doubtless that LARP1 is correlated with high malignancy and poor prognosis in most cancers. Zhang et al. [56] found that miR-129-5p can directly bind to LARP1 and lncRNA KCNQ1OT1 and inhibit the progression of cell proliferation, invasion, and drug resistance when KCNQ1OT1 expression is suppressed.

### 5.2. Interaction with mRNA

Among all kinds of RNAs, mRNAs are the most common corresponding factors that bind with RBPs through 3′ or 5′-UTRs or coding sequences and take part in posttranscriptional regulation [109]. The emerging roles of mRNA-RBP complexity in regulating malignant cancers show a bright new way to treat cancer, and the discoveries of complexity in OS are summarized below.

As confirmed in 1995 for the first time [110], RNA-binding motif protein 10 (RBM10) is an RBP containing two RRMs and is located at p11.23 on the X chromosome. RBM10 is regarded as a tumor suppressor [111–113], but recent studies have shown that in small cell lung cancer, RBM10 has a reversed exertion with endogenous RBM5 deletion [114]. The dual state makes RBM10 an intricate therapeutic target, and its mechanism in OS is unknown. Han et al. [30] investigated the role of RBM10 in OS and found that the efficiency of RBM10 upregulation was a feasible avenue for inhibiting OS growth. During the exploration of material inhibition, downregulated Bcl-2 and upregulated caspase-3, and TNF-*α* mRNA were selected as potential regulators to induce cell apoptosis.

Xu et al. [32] affirmed that HuR binds directly to YAP and showed its promotor role by YAP expression. Further study detected the sensitivity development of adriamycin in OS cells by HuR knockout and YAP dependence (Figure 2(c)).

The MSI RBPs were first found in Drosophila by Nakamura et al. [115], and humans have two homologs, Musashi-1 (MSI1) and Musashi-2 (MSI2), consisting of two RRMs [65]. *MSI1* is a kind of stem cell-related gene that is involved in early asymmetric divisions generating differentiated cells from neural stem cells or progenitor cells. MSI1 can be upregulated in multiple cancers [116–118] and was elucidated as a prognostic factor in breast cancer and ovarian cancer [119, 120]. Niu et al. [36] obtained evidence showing the appearance of increased expression of MSI1 in OS and proved the inhibition of MSI1 knockdown on proliferation, apoptosis, tumor formation, and cell cycle arrest of OS cells. Then, they found the relationship between knockdown MSI1 and increment of p21 and p27 protein that indicated the effect of MSI1 on cell cycle arrest.

Al-Khalaf and Aboussekhra [39] hypothesized that the combination of AUF1 with VEGF-A mRNA and HIF-1*α* mRNA was correlated with controlling angiogenesis. AUF1 was highly expressed in OS cells and was demonstrated to induce VEGF-A and HIF-1*α*, which are typical factors in hypoxic conditions. Thus, antiangiogenic therapies targeting AUF1 could provide effective methods for treating OS.

IGF2BP3 shares 73% amino acid sequence identity with IGF2BP1 and is particularly interesting in tumorigenesis and tumor progression. Compared to human tissue, the expression of IGF2BP3 is highly elevated in stomach adenocarcinoma, skin cutaneous melanoma, lung adenocarcinoma, etc. [121]. Previously, IGF2BP3 was shown to predict metastasis and angiogenic potential in human OS [122, 123]. In a subsequent study, IGF2BP3 could regulate mRNA lgf2 to affect cell tumorigenicity in vivo in murine AXT cells [48], but data analyses refused to affirm the single contribution of lgf2 in regulation, thus leaving further experiments to uncover the mechanism.

TARBPs are a group of RBPs with typical double-stranded RNA-binding domains (dsRBDs). The dsRBD consists of 70–90 amino acids and a characterized *αβββα* protein folding pattern to recognize dsRNA [124, 125] and has RNA interference, localization, editing, and translocation abilities [65]. TARBP2, the branch of TARBP, acts as an RNA-binding subunit on the RNA-induced silencing complex and is capable of influencing miRNA processing and maturation [126, 127]. Notably, overexpressed or downregulated TARBP2 was found in various cancers [128–130], and the assumption of improvement in tumor angiogenesis and metastasis was verified through dysregulating miRNAs in lung, breast, and liver cancer cell lines [131]. In OS cells, TARBP2 was observed to have decreased expression and target the anticancer gene *let-7f-5p* [49]. The regulation between TARBP2 and *let-7f-5p* is negative, where TARBP2 can activate *let-7f-5p*, and *let-7f-5p* will also downregulate TARBP2 in return. In particular, researchers have pointed out a feedback loop consisting of TARBP2 and *let-7f-5p* that strongly modulates the two genes to a low level of expression and is induced by hypoxia. The feedback loop was demonstrated to hamper the activation of the Wnt signaling pathway so that the proliferation and invasion of OS cells were largely promoted.

The expression of lncRNA DDX11 antisense RNA 1 (DDX11-AS1) was found to be elevated in OS cells, and Zhang et al. [47] demonstrated the contribution of DDX11-AS1 to OS cell progression by regulating the RBP DDX11. IGF2BP2 was consistent with its oncogenic role in OS and bound to DDX11-AS1 to upregulate DDX11 expression, whereas the anticancer gene *miR-873-5p* was attenuated by DDX11-AS1 to reach the same goal.

### 5.3. Interaction with Other Types of Molecules

hnRNPK is considered a DNA/RNA binding protein as well as a coregulator of p53 participating in RNA splicing, mRNA stabilization, translation, chromatin restructuring, and the DNA damage response [132, 133]. In particular, high expression of hnRNPK is observed in several cancers, and its promoting effect on cell metastasis and its significance in poor prognosis implicate hnRNPK as a cancer promotor [134]. However, arginine-methylated hnRNPK has a negative impact on OS cells by hampering the interaction between DDX3 and hnRNPK. Similarly, inhibition of DDX3 helicase promoted cell apoptosis, and this characteristic belonged to RK-33 [42]. Research gives us a bright new decoy to OS cell apoptosis.

Heterogeneous nuclear ribonucleoprotein A1 (hnRNPA1) is the hub factor involved in pre-mRNA processing and RNA splicing [135]. FASN is a lipogenic enzyme that contains two polypeptides. Both of these polypeptides are characterized as oncogenes that promote cancer cell progression in several cancers. An initial study elucidated that FANS activates the PI3K/Akt signaling pathway to induce OS cell migration and invasion [136], but the latest research [52] validated that hnRNPA1 was the downstream target of FANS and was positively related to FANS expression. Elevated hnRNPA1 expression was associated with poor prognosis in OS and proliferation, migration, and invasion of OS cells. The therapeutic value of hnRNPA1 deserves to be explored in further studies.

## 6. Therapeutic Value of RBPs in OS

Generally, cancers are too complex to explore the complete reason why we have no specific treatments for curing various cancers at present. OS is a troublesome challenge that hurts doctors all over the world due to its rarity, complexity, and difficulty. Clinical therapies have significantly improved thanks to the amazing inspiration of combining surgical operation and radiotherapy with neoadjuvant and adjuvant chemotherapy but have failed to renew for a long time. Many articles about sensitive biomarkers have manifested their favorable function in distinguishing the molecular differences in specific tumors and are beneficial to adjust the present treatment strategy. RBPs, with enrichment in biological processes, have been shown to have prognostic and pharmacological value in OS in recent years.

### 6.1. Evidence of RBPs as Prognostic Factors in OS

**Figure 3:**
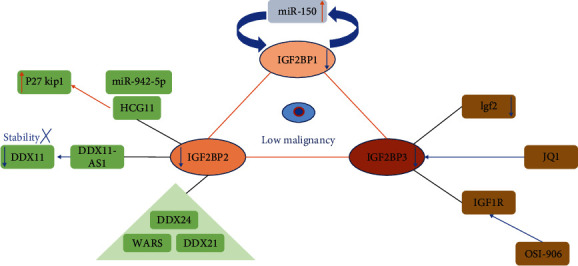
Multiple functions of IGF2BPs in reducing OS malignancy: regulatory mechanism, medicine therapy, and prognosis. Red arrows represent positive regulation, while blue ones are negative.

The prognostic value of RBPs has been recognized in recent years when RBPs and their associated molecules were considered simultaneously, which will help us renew the understanding of different tumor recoveries. The IGFBP family plays irreplaceable roles in this section (Figure 3). Statistical data from 100 patients with OS showed that miR150/IGF2BP1 expression (*P* = 0.01, for overall/disease-free survival, Cox proportional hazard model) was a symbolic target for poor response in OS therapy. Kaplan–Meier and log-rank tests suggested that the expression levels of two targets resulted in four degrees of recovery, in which low miR-150 and high IGF2BP1 expression were the worst [87].

In addition to predicting patients' recoveries by means of both RBPs and their correlated molecules, systematic analysis is another high-efficiency strategy to select target RBPs. In the beginning of 2021, Li et al. [137] published the results that four key RBPs (DDX24, DDX21, WARS, and IGF2BP2) were identified as prognostic factors in OS. GSE33382, a GEO dataset of OS, first revealed 38 RBPs aberrantly expressed from the comparison between tumor and normal samples, and then enrichment analysis and PPI network analysis were performed to predict the potential functions and connections of these RBPs. Subsequently, univariate and multiple stepwise Cox regression analyses selected DDX24, DDX21, WARS, and IGF2BP2 as hub RBPs, among which WARS was closely correlated with tumor immune infiltration. A risk score and a prognostic model were constructed, and the calculation suggested that both have the expected properties. At the end of 2021, Zhang et al. [138] developed and validated another 10 RBPs (TDRD6, TLR8, NXT2, EIF4E3, RPS27L, CPEB3, RBM34, TERT, RPS29, and ZC3HAV1) as novel predictive factors in OS. With the analyses, the risk assessment model was prominent in connection with metastasis, and the nomogram is the dependable strategy to predict patient survival, indicating that the prognostic function of RBPs has a great influence in the future.

### 6.2. RBPs in Medicine Therapy

Apart from the prognostic value in predicting OS malignancy, what cannot be ignored is the applications of RBPs in pharmacology existing underlying remedies. High expression of IGF2BP3 can be decreased by the inhibitor JQ1 [139], but low expression of IGF2BP3/IGF1R is sensitive to the inhibitor OSI-906, which specifically targets IGF1R but not IGF2BP3 [48]. Not only inhibitors but also medicines that act on RBPs make researchers continually update pharmacologic ways to reduce tumor cell viability. Some medicines were certified to adapt RBP expression. Knockdown of DDX5 has been reported to be able to moderate camptothecin (CPT)-induced DNA damage, and the interaction protein NONO was observed to coimmunoprecipitate with DDX5. Thus, camptothecin can restore the cpt resistance of OS by breaking down the bond of DDX5 and NONO at the protein level [140]. Adriamycin was either observed to be sensitive to OS cells with HuR or HuR-related lncRNA knockdown [32, 33]. In summary, RBPs have been extensively used in exploring therapeutic strategies in the pharmacology of OS.

## 7. Perspectives

RBPs are critical regulators that participate in OS cell progression by controlling mRNAs, lncRNA-miRNA complexes, and RBPs or impacting other signaling pathways. However, as the understanding of RBPs in OS gradually accumulates, research on the therapeutic value of RBPs is still too preliminary to certify its contribution in prognosis and therapy strategies. Compared to the numerous RBPs identified, only some of them were validated to have an abnormal expression in cancers, let alone rare tumors such as OS. Therefore, updated certifications on new oncogenic or nononcogenic abilities of RBPs deserve more attention. Additionally, the conclusions in Table 1 are consistent with the notion that RBPs exert positive or negative effects on OS; thus, inhibitors of molecules or techniques, such as the CRISPR-Cas9 system, directly regulating RBP expression or inhibiting RBP function indirectly can be possible ways to suppress OS cell progression. In medicine therapy, drug-resistance cannot be avoided, but the key to solve the problem can be another succedaneum or specific structure to improve the efficiency. Similar to adriamycin, verteporfin is another YAP-TEAD binding inhibitor that can repress OS cell progression by reducing YAP expression and activation in transcription, suggesting its possible application in curing adriamycin-resistant patients, but more reliable data need to be explored in future studies. Estrogen-related receptor alpha was found to employ IGF2BP1 to facilitate mRNA stability and rescue the efficiency of adriamycin in chemotherapy-resistant OS cells [141]. At present, attempts to uncover the characteristics of RBPs in DNA damage and radiotherapy broaden our insights into OS treatment. DNA damage is one method to stimulate cell apoptosis, and RBM10 [30] and hnRNPK with arginine methylation [42] were proven to play support or suppression roles in this progress. Previous studies have identified five correlating RBPs (RUNX2, CDC5L, MDM2, RECQL4, and CDK4) as new clues in pediatric OS for chemotherapy-negative response [142], but further experiments are needed to explain the underlying principles. Radiotherapy uses radiation or X-rays to kill tumor cells, and the efficacy is largely correlated with the oxygen level. Hypoxia fails to stabilize radiation to produce DNA radicals and causes DNA damage that prevents tumor cells from death [143]. Additionally, hypoxia was found to have a positive influence on RBP overexpression. Decreased expression of the interaction loop of TARBP2 and let-7f-5p was verified under hypoxic conditions [49]. To prevent hypoxia and improve radiotherapy effects, bacteria have the potential as a medium. As probiotics, bacteria benefit the host, and many studies have attested to their ability to enhance the radiotherapy effect under hypoxia and protect normal tissue [144]. Taken together, we suggest the development potential of RBPs in OS treatment.

## 8. Conclusions

RBPs are proteins that bind to multiple RNAs and have been identified as potential targets in cancers by modulating cancer cell proliferation, migration and invasion, apoptosis, and EMT. RBPs have identified complex characteristics in various cancers, and dual effects in regulating OS have recently been discovered. Summarizing previous studies, we naturally concluded that HuR, AUF1, PTBP1, and IGF2BPs were confirmed to promote OS cell progression, while TARBP2, PUM2, QKI2, and RBM10 acted as suppression factors. Of note, the close relationship between RBPs and binding molecules helped us comprehend the mechanism of RBP regulation. mRNA, miRNAs, and lncRNAs have gradually manifested considerable roles in OS by combining and influencing RBPs. Anticancer miRNAs and oncogenic lncRNAs are canonical couples in regulating cancers, and RBPs have dramatic joint mediation on the couples in OS, thus suggesting an indirect way to affect RBP function. Additionally, accumulating evidence has shown the prognostic value of RBPs that are able to distinguish tumor malignancy, and inhibitors or medicines targeting RBPs exert favorable efficiency on OS treatment. However, the existing findings are too small to completely explain the whole mechanism. What can be ascertained is that new avenues to enrich our knowledge of OS are visible in studying RBPs, and new methods to attenuate tumor malignancy are feasible through modulating RBPs or their binding. The fruitful achievements we can see in this regard are forthcoming.

## Figures and Tables

**Table 1:** Summary of RBPs regulating OS.

Sarcoma types	RBD	RBPs	Expression in OS	Protein function	Targets	Signaling pathway or axis	Cell lines	Reference
Osteosarcoma	RRM	RBM10	–	Alternative splicing, 3′- end processing of some pre-mRNAs, p53 stabilization, cell cycle arrest, and antiviral reactions	Bcl-2, caspase-3, and TNF-*α*	N	U2OS	[30]
		HuR	+	mRNAs stability, translation, and nucleus-to-cytoplasm shuttling	AGO2, lncRNA xist	lncRNA XIST/AGO2	hFOB1.19	[31]
					YAP	N	HEK293T, SaOS2, 143B, U2OS, MG63	[32]
					LncRNA B4GALT1-AS1	B4GALT1-AS1/HuR/YAP axis	MG63, U2OS, Saos2, 143B	[33]
					DUXAP10, SOX18	N	U2OS, SAOS2, HOS, MG63, hFOB1.19	[34]
					HMGA1	miR 142 3p/HMGA1 axis	MG63	[35]
		MSI1	+	Regulation of early asymmetric divisions	p21 and p27	N	MG63, HOS	[36]
		MSI2	+	Asymmetric division, hematopoietic, and intestinal systems, self-renew	miR-149, LncRNA DANCR	miR-149/MSI2 axis	hFOB1.19, Saos-2	[37]
		AUF1	+	mRNA stability and mRNA translation	miR-141 and miR-146b-5p	N	U2OS, HOS, MG63, 143B, SaOS2, HFSN1	[38]
					HIF-1*α* and VEGF-A	N	U2OS, HOS, MG63, 143B	[39]
		PTBP1	+	mRNA splicing, translation, stability, and localization	LncRNA HOTTIP	Wnt/*β*-catenin signaling pathway	hFOB 1.19, U2OS, MG63, Saos-2	[40]
	Pumilio family	PUM2	–	mRNA repression; mRNA degradation	miR-590-3p and miR-9	RhoA/Rock pathway	MG63, Saos2	[41]
	KH domain	hnRNPK	+	RNA splicing, mRNA stabilization, translation, chromatin restruction, and DNA damage response	DDX3	N	U2OS	[42]
		QKI2	–	Facilitating spliceosomal complex formation, mRNA stability, and localization	miR-17-92 cluster	miR-17-92 cluster/QKI2/*β*-catenin axis	hFOB 1.19, HOS, 143B, HEK-293TN	[43]
					miR-20a	N	293TN, 143B, hFOB 1.19	[44]
	RRM&KH domain	IGF2BP1	+	mRNA stability, translatability, or localization	miR-150	miR-150- IGF2BP1 axis	N	[45]
		IGF2BP2	+	RNA localization, stability, and translation, abate RNAs endonucleases or microRNA-mediated degradation	lncRNA HCG11	N	MG63, U2OS, HOS, 143B, Saos-2, hFOB1.19	[46]
					DDX11-AS1	N	SaOS-2, MG-63, HOS, U2OS, hFOB1.19	[47]
		IGF2BP3	+	mRNA stability, localization	Igf2	N	AXT, AX	[48]
	dsRBD	TARBP2	–	miRNA processing and maturation	let-7f-5p	Wnt signaling pathway	Hfob, U2OS, HOS, Saos	[49]
	PrLD	FUS	+	RNA splicing	miR-141-3p,LDHB	N	U2OS, Saos-2, 143B, MG-63, HOS, hFOB	[50]
		EWSR1	+	Transcription and RNA splicing	miR-199a-5p, Sox2	miR-199a-5p/Sox2 axis	MG63	[51]
		hnRNPA1	+	Pre-mRNA processes	Fatty acid synthase	Fatty acid synthase /hnRNPA1	hFOB1.19, 143B, HOS, U2OS	[52]
	DEAD-box	DDX5	+	Translation, RNA decay, and miRNA processing	miR-671-5p	miR-671-5p/DDX5 axis	hFOB1.19, HOS, MG63, U2OS, and Saos-2	[53]
					miR-214-5p	circ-XPR1/miR-214-5p/DDX5 axis	hFOB1.19, U2OS, U2OS/MTX300, HOS, MG63, 143B, ZOS, and ZOSM	[54]
	Cap-binding	eIF4E	+	RNA export, translation, RNA stability. and/or sequestration	miR-496	N	Saos-2, HOS, U-2OS, SOSP-9607, MG63, hFOB1.19	[55]
	La module	LARP1	+	mRNA stability and mRNA translation	miR-129-5p	miR-129-5p/LARP1 axis	HFOB1.19, HOS, MG63, 143B,U2OS	[56]
	RRM&Zinc finger domain	CPEB1	+	mRNA translation	miR-320a	N	143B, U2OS, hFOB	[57]
					miR-377-3p	miR-377-3p/CPEB1 axis,	N	[58]
		CPEB4	+	Polyadenylation, mRNA translation	lncRNA RP11-361F15.2, miR-30c-5p	RP11-361F15.2/miR-30c-5p/CPEB4 loop	MG-63, U2OS, HOS, 13B, hFOB 1.19, RAW264.7	[59]

“+” represents upregulated. “−” represents downregulated. “N” not detected.

## Data Availability

Data are available upon reasonable request to the corresponding author.

## Abbreviations

OS: Osteosarcoma
RBPs: RNA-binding proteins
EFS: Event-free survival
EMT: Epithelial-mesenchymal transcription
RBDs: RNA-binding domains
RRM: RNA recognition motif
KH: K homology
PrLD: Prion-like domain
m7GpppN: N7 methylated guanine
3′-UTR: 3′-Untranslated region
MSI: Musashi
PCBP1: PolyC-RNA-binding protein 1
RBM38: RNA-binding motif protein
IGF2BP3: IGF2 mRNA-binding protein 3
HuR: Human antigen R
hnRNPK: Heterogeneous nuclear ribonucleoprotein K
miRNAs: MicroRNAs
AUF1: AU-binding factor 1
pre-mRNA: Premessenger RNA
PUM: Pumilio
QKI: Quaking
STAR: Signal transduction and activation of RNA
FUS: Fused in sarcoma
ALS: Amyotrophic lateral sclerosis
LDHB: Lactate dehydrogenase B
EWSR1: Ewing sarcoma breakpoint region 1
DEAD: Asp-Glu-Ala-Asp
eIF4E: Eukaryotic translation initiation factor 4E
CPEB: Cytoplasmic polyadenylation element-binding protein
lncRNAs: Long noncoding RNAs
DUXAP10: Double homeobox A pseudogene 10
DANCR: Anti-differentiating noncoding RNA
PTBP1: Polypyrimidine tract-binding protein 1
LARP1: La-related protein 1
RBM10: RNA-binding motif protein 10
MS1-1: Musashi-1
MSI-2: Musashi-2
dsRBDs: Double-stranded RNA-binding domains
RISC: RNA-induced silencing complex
DDX11-AS1: DDX11 antisense RNA 1
hnRNPA1: Heterogeneous nuclear ribonucleoprotein A1
CPT: Camptothecin
ERR*α*: Estrogen-related receptor alpha.
